# Effects of Xipayi Gingival Solution combined with minocycline hydrochloride on inflammatory cytokines and masticatory function in patients with chronic periodontitis

**DOI:** 10.3389/fcimb.2026.1739777

**Published:** 2026-02-25

**Authors:** Meijie Shen, Yanjie Chai, Shenxi Yao, Lijie Xu

**Affiliations:** Department of Stomatology, Tongxiang First People’s Hospital, Tongxiang, Zhejiang, China

**Keywords:** chronic periodontitis, inflammatory factors, masticatory function, minocycline hydrochloride, Xipayi Gingival Solution

## Abstract

**Objective:**

This study aimed to elucidate the therapeutic effects of Xipayi Gingival Solution (Xipayi Guyin Ye) combined with minocycline hydrochloride on inflammatory factors and masticatory function in patients with chronic periodontitis.

**Methods:**

Ninety-eight patients with chronic periodontitis were randomly allocated into two groups. Both groups underwent conventional periodontal therapy. The control group received minocycline hydrochloride once weekly for four weeks, while the combination group additionally used Xipayi Gingival Solution three times daily for four consecutive weeks. Periodontal health indicators (PD, AL, PLI, SBI, GI, GCFV), gingival crevicular fluid cytokines (hs-CRP, TNF-α, IL-1β, IL-6), serum oxidative stress markers (MDA, SOD, GSH-Px), masticatory function (CFQ), pain (VAS), clinical efficacy, and adverse reactions were compared.

**Results:**

Four control and two combination group patients withdrew. Finally, 45 and 47 patients completed treatment. Post-treatment, PD, AL, PLI, SBI, GI, and GCFV decreased in both groups, with lower values in the combination group (*P* < 0.05). Inflammatory and oxidative stress markers improved more in the combination group (*P* < 0.05). CFQ scores increased and VAS scores decreased in both groups, with better outcomes in the combination group (*P* < 0.05). Total effective rate was higher in the combination group (97.87% vs 84.44%, *P* < 0.05), while adverse reactions were comparable (8.51% vs 4.26%, *P* > 0.05).

**Conclusion:**

Xipayi Gingival Solution combined with minocycline hydrochloride enhances periodontal status, mitigates inflammatory and oxidative stress responses, improves masticatory function, and relieves pain without increasing adverse events. The combined therapy demonstrates better clinical efficacy than minocycline alone, indicating a safe and effective therapeutic approach for chronic periodontitis.

## Introduction

Chronic periodontitis is a prevalent inflammatory disease characterized by polymicrobial infection and progressive destruction of periodontal supporting tissues. Its incidence has increased steadily in recent years (Gao et al., 2021). Dental plaque microorganisms are the main etiologic agents, contributing to the breakdown of gingiva, periodontal ligament, cementum, and alveolar bone (Lasica et al., 2024). Conventional management focuses on removing plaque and calculus, often supplemented with locally delivered antimicrobials such as tetracycline, metronidazole, minocycline, doxycycline, or chlorhexidine to control infection and inflammation (Gandhi et al., 2025). Therefore, local adjunctive drug therapy has emerged as an important management strategy aimed at directly acting on the periodontal microenvironment to enhance infection control and modulate inflammatory responses (Martinez-Garcia and Hernandez-Lemus, 2025).

The fundamental rationale for local drug delivery in the treatment of chronic periodontitis lies in its ability to directly deliver high concentrations of drugs to the affected site (periodontal pockets), thereby achieving effective killing or inhibition of pathogenic microorganisms while minimizing systemic absorption and its associated potential side effects (Vivek et al., 2025). Local drug administration is typically used as an adjunct to basic periodontal treatment (such as supragingival and subgingival scaling) for moderate to severe chronic periodontitis (Vivek et al., 2025). Its clinical benefits are primarily manifested in enhanced clearance of key periodontal pathogens and significant improvements in clinical indicators, such as reduced periodontal probing depth (PD), decreased bleeding on probing rates, and promotion of clinical attachment level (AL) gain (de Sousa et al., 2021; Gandhi et al., 2025; Vivek et al., 2025).

Minocycline hydrochloride is one of the representative local drugs in this category. As a broad-spectrum tetracycline derivative, minocycline hydrochloride exerts both antibacterial and anti-inflammatory actions in periodontitis (Yang et al., 2022). It modulates cytokine activity, alleviates inflammation and pain (Chugh et al., 2022) and effectively suppresses key periodontal pathogens such as *Porphyromonas gingivalis*, *Prevotella intermedia*, *Fusobacterium nucleatum*, and *Actinomyces* species (Zeng et al., 2024). However, as a broad-spectrum antibiotic, it may disrupt the microbial balance in the oral environment, leading to dysbiosis (Chen et al., 2020). Xipayi Gingival Solution (Xipayi Guyin Ye, also referred to as Xipaiyigu Gingival Rinse in previous studies), a tea polyphenol–based preparation derived from Uyghur medicine, exhibits strong antibacterial, antiviral, and antioxidant effects and is widely used for gingival diseases and oral ulcers (Mai et al., 2021). Its core compound, tannin (a complex polyphenolic compound), possesses traditional effects such as tissue astringency, enhancement of local resistance, and hemostasis (Chen et al., 2020; Chen et al., 2024). Modern research indicates that the clinical efficacy of the solution primarily stems from its significant anti-inflammatory effects, sustained antibacterial activity, and ability to regulate oral microecological balance (Chen et al., 2020).

Based on this, Xipayi Gingival Solution theoretically complements the limitations of pure antibacterial therapy by more comprehensively regulating the imbalanced periodontal microenvironment through its antioxidant and anti-inflammatory properties. The combined use of Xipayi Gingival Solution and minocycline hydrochloride has a clear scientific basis: the former, through the antioxidant, anti-inflammatory, and broad-spectrum antibacterial effects of polyphenolic compounds (Chen et al., 2020; Mai et al., 2021; Chen et al., 2024), disrupts biofilm structure and alleviates oxidative damage; the latter, through its specific antibiotic action and enzyme inhibitory effects, targets and kills pathogenic bacteria while inhibiting tissue degradation (Chen et al., 2020; Chugh et al., 2022; Yang et al., 2022; Zeng et al., 2024). The combination of the two may produce a synergistic effect, more effectively controlling infection, breaking the inflammatory cycle, reducing tissue destruction, and potentially promoting periodontal tissue repair and functional recovery through different pathways. However, rigorous clinical studies are currently lacking to systematically evaluate the impact of this combined therapy on key pathophysiological indicators and important functional outcomes (such as chewing ability) in patients with chronic periodontitis.

Therefore, this study was conducted to systematically evaluate the efficacy of Xipayi Gingival Solution (Xipayi Guyin Ye) combined with minocycline hydrochloride in patients with chronic periodontitis, focusing on its effects on inflammatory cytokines, oxidative stress markers, and masticatory function. The findings aim to provide new evidence supporting an integrated local treatment strategy.

## Materials and methods

### Ethical approval

This clinical study was reviewed and approved by the Ethics Committee of Tongxiang First People’s Hospital, and all participants provided written informed consent prior to enrollment.

### Sample size calculation

High-sensitivity C-reactive protein (hs-CRP) in gingival crevicular fluid (GCF) was utilized as the primary outcome. Based on preliminary results, the mean ± standard deviation of hs-CRP was 9.13 ± 2.97 μmol/L in the combination group and 11.28 ± 3.11 μmol/L in the control group. Sample size was calculated using the formula for comparing two independent means ( $n={\left[\frac{(Z_{\alpha /2}+Z_{\beta })\sigma }{\delta }\right]}^{2}(Q_{1}^{-1}+Q_{2}^{-1})$), with a two-sided α = 0.05 and statistical power (1–β) = 0.90. The calculation yielded approximately 42 participants per group. Considering a potential 15% dropout rate, 49 participants were recruited per group, totaling 98 subjects to ensure sufficient power.

### General information

Ninety-eight patients with chronic periodontitis admitted to Tongxiang First People’s Hospital from October 2024 to June 2025 were enrolled and randomly assigned into a control group or a combination group (49 each) using a random number table. Baseline characteristics, including sex, age, periodontitis stage, smoking history, and educational level, presented no differences between the two groups (*P* > 0.05), indicating comparability (**Table 1**).

**Table 1:** Comparison of general characteristics between the two groups.

Indicator	Combination group (n = 49)	Control group (n = 49)	χ^2^/t	*P*
Sex			0.163	0.686
Male	26 (53.06)	24 (48.98)	–	–
Female	23 (46.94)	25 (51.02)	–	–
Age (years)	35.00 ± 7.00	34.24 ± 7.38	0.52	0.604
Periodontitis stage			0.167	0.683
Stage II	27 (55.10)	29 (59.18)	–	–
Stage III	22 (44.90)	20 (40.82)	–	–
Smoking history			0.044	0.833
No	31 (63.27)	32 (65.31)	–	–
Yes	18 (36.73)	17 (34.69)	–	–
Education level			1.684	0.431
Junior high school or below	14 (28.57)	20 (40.82)	–	–
High school	18 (36.73)	14 (28.57)	–	–
College or above	17 (34.69)	15 (30.61)	–	–

### Inclusion criteria

① Patients diagnosed with stage II or III chronic periodontitis according to the *2018 Classification of Periodontal and Peri-implant Diseases*; ② Aged 18–65 years, any sex, full consciousness, and able to comply with treatment and follow-up; ③ First-time visit to our department, with no prior basic periodontal therapy (scaling, subgingival curettage, etc.) or use of tetracyclines (e.g., minocycline) or local periodontal medications (e.g., Xipayi Gingival Solution) within the previous three months; ④ With at least 20 remaining permanent teeth [Selection criteria for target teeth: For a single affected tooth, the PD of the periodontal pocket ≥ 5 mm, clinical attachment loss ≥ 3 mm, and whole-mouth bleeding on probing > 30%; tooth mobility of the affected tooth ≤ Grade II; no pulp necrosis, apical periodontitis, or Grade III-IV furcation involvement; no extensive tooth defects or irritation from the margins of poor restorations]; ⑤ No systemic use of glucocorticoids, immunosuppressants, or non-steroidal anti-inflammatory drugs (e.g., ibuprofen, aspirin) within three months to avoid interference with inflammatory factor measurements; ⑥ No oral mucosal lesions such as ulcers or herpes.

### Exclusion criteria

① Patients with other forms of periodontal diseases, such as aggressive periodontitis or reactive proliferative gingivitis; ② Pregnancy, lactation, or planned pregnancy; ③ Allergy to minocycline hydrochloride, any component of Xipayi Gingival Solution, or tetracycline hypersensitivity history; ④ Systemic diseases severely affecting periodontal tissues, such as uncontrolled diabetes, osteoporosis, thyroid dysfunction, hematologic or immune disorders; ⑤ Severe cardiac, hepatic, or renal dysfunction, or malignancy; ⑥ Oral surgery within the past six months, such as flap surgery or dental implantation, or planned surgery during the study period; ⑦ Long-term use (≥ 6 months) of medications potentially affecting periodontal metabolism or healing, such as calcium-channel blockers, phenytoin, or immunosuppressants; ⑧ Extremely poor oral hygiene unamenable to improvement, or psychiatric/cognitive disorders impairing compliance; ⑨ Participation in another clinical trial or recent (within one month) use of agents with similar antimicrobial mechanisms (e.g., metronidazole, tinidazole).

### Withdrawal and dropout criteria

① Participants found after enrollment to no longer meet inclusion criteria or to have previously undetected exclusion criteria; ② Poor adherence during treatment or occurrence of severe adverse events or complications during follow-up, such as severe drug allergy or secondary severe infection, requiring treatment discontinuation as assessed by investigators; ③ Use of prohibited medications during the study, such as tetracycline antibiotics or non-steroidal anti-inflammatory drugs, or receipt of additional periodontal therapies that could interfere with outcome assessment; ④ Voluntary withdrawal for personal reasons or loss to follow-up resulting in missing key data.

## Methods

All patients in both groups first received standardized basic periodontal treatment performed by the same experienced attending dentist over a four-week period. The treatment procedures included: ① Oral hygiene instruction: Patients were thoroughly educated on the Bass brushing technique and advised to use dental floss or interdental brushes for daily plaque control; ② Supragingival scaling: Using an ultrasonic scaler (manufacturer: Satelec, France; model: P5 NEWT), plaque, calculus, and surface stains were completely removed from tooth crowns; ③ Subgingival curettage and root planning: Under local anesthesia, a Gracey curette (manufacturer: Hu-Friedy, USA) was employed to thoroughly remove subgingival plaque and calculus from periodontal pockets and to smooth the root surfaces.

On this basis, the control group received topical minocycline hydrochloride ointment (Manufacturer: Sunstar Inc., Japan; Approval No.: HJ20150106; Specification: 0.5 g per tube). Medication was applied once weekly by the physician for four consecutive weeks. Procedure: The periodontal pocket of the target tooth was isolated and dried, then the syringe tip of the ointment was carefully inserted to the base of the pocket, and the gel was injected slowly until the pocket was filled and a small overflow was visible. Patients were instructed to refrain from rinsing, drinking, or eating for one hour after application.

The combination group received the same minocycline hydrochloride regimen as the control group, together with Xipayi Gingival Solution (Xipayi Guyin Ye; Manufacturer: Xinqikang Pharmaceutical Co., Ltd.; Approval No.: Z65020012; Specification: 30 mL per bottle). The rinse was used three times daily—after breakfast, lunch, and dinner—for four weeks. Each time, approximately 3–5 mL of the undiluted solution was held in the mouth and swished for 2–3 minutes before swallowing (no significant or persistent staining will occur). During treatment, patients were advised to avoid smoking, alcohol consumption, and spicy foods.

### Outcome measures

1. All periodontal examinations were performed by the same senior clinician who was unaware of the patients’ group assignments, using a Williams periodontal probe to ensure consistency. The following indices were assessed before treatment (the day before the start of drug intervention) and after treatment (the first day after the completion of the four-week drug intervention): ① PD: The distance from the gingival margin to the pocket base was measured under a probing force of 0.20–0.25 N at six sites per tooth (mesiobuccal, midbuccal, distobuccal, mesiolingual, midlingual, and distolingual), and the mean value was recorded; ② AL: Determined as the distance between the cementoenamel junction and the pocket base; ③ Plaque Index (PLI): Evaluated using the Löe–Silness index: 0 = no plaque; 1 = thin film removable with a probe; 2 = moderate plaque at the gingival margin or sulcus; 3 = heavy soft deposits (Loe and Silness, 1963); ④ Sulcus Bleeding Index (SBI): Recorded 30 s after probing as 0 = none, 1 = punctate bleeding, 2 = bleeding along the margin, or 3 = profuse bleeding (Muhlemann and Son, 1971); ⑤ Gingival Index (GI): Scored following the Löe–Silness system: 0 = healthy; 1 = mild inflammation without bleeding; 2 = moderate inflammation with redness and bleeding; 3 = severe inflammation with marked swelling, ulceration, or spontaneous bleeding (Loe and Silness, 1963); ⑥ Gingival Crevicular Fluid Volume (GCFV): Measured using Whatman #1 filter paper strips (2 mm × 10 mm) inserted into the sulcus for 30 s, and quantified with an electronic GCF analyzer.
2. Inflammatory cytokines in GCF: GCF samples were obtained before treatment (the day before the start of drug intervention) and after treatment (the first day after the completion of the four-week drug intervention), using filter paper strips. The absorbed fluid was eluted by centrifugation, and concentrations of hs-CRP, TNF-α, IL-1β, and IL-6 levels were quantified using ELISA kits as per the manufacturer’s instructions (Shanghai ELISA Biotechnology Co., Ltd.).
3. Oxidative stress indicators: Fasting venous blood samples (4 mL) were obtained from each participant before treatment (the day before the start of drug intervention) and after treatment (the first day after the completion of the four-week drug intervention). Samples were centrifuged at 3000 r/min for 10 min to separate serum. Superoxide dismutase (SOD) activity was determined using the xanthine oxidase method, malondialdehyde (MDA) levels were measured by the thiobarbituric acid assay, and glutathione peroxidase (GSH-Px) activity was evaluated by a colorimetric method. All analyses were conducted following the instructions provided in the commercial assay kits (Nanjing Jiancheng Bioengineering Institute, China).
4. Chewing function: Chewing performance was evaluated using the Chinese version of the Chewing Function Questionnaire (CFQ) (Fan et al., 2021) before treatment (the day before the start of drug intervention) and after treatment (the first day after the completion of the four-week drug intervention). The questionnaire includes 10 commonly consumed food items classified into five difficulty levels (L1–L5), with two representative foods and three substitutes per level. For each item, participants selected one of three options—”unable to chew,” “difficult to chew,” or “easy to chew”—scored 1, 2, or 3 points, respectively. The total score ranged from 10 to 30, with higher values indicating better chewing ability. The instrument demonstrates high internal consistency (Cronbach’s α = 0.912), excellent sampling adequacy (KMO = 0.939), and reliable test–retest stability.
5. Pain assessment: Pain intensity was assessed using a 10-cm Visual Analogue Scale (VAS) (Huskisson, 1974) before treatment (the day before the start of drug intervention) and after treatment (the first day after the completion of the four-week drug intervention), with endpoints labeled “no pain” (0) and “worst imaginable pain” (10). Participants marked their perceived pain level on the line, and the distance from the left end was recorded as the VAS score.
6. Clinical efficacy evaluation: Clinical efficacy was determined after treatment (the first day after the completion of the four-week drug intervention) according to the *Guidelines for the Diagnosis and Treatment of Oral Diseases*. ① Markedly effective: PD reduced by ≥ 3 mm, AL decreased by ≥ 50%, teeth stable, gingival pain and swelling completely resolved; ② Effective: PD reduced by 1–2 mm, AL and tooth mobility improved, gingival symptoms mostly relieved; ③ Ineffective: No obvious improvement in PD, AL, tooth mobility, or gingival inflammation. Total effective rate = (markedly effective + effective cases)/total cases × 100%.
7. Adverse reactions: All adverse events during treatment were documented, including oral mucosal irritation, taste alteration, tooth sensitivity, and allergic responses.

### Statistical analysis

Data analysis was carried out using SPSS software (version 27.0; IBM, USA). All tests were two-sided, and statistical significance was established at *P* < 0.05. The distributional characteristics of quantitative data were examined with the Shapiro–Wilk test. Variables not conforming to normality were described as median (P25, P75). Comparisons between groups were conducted with the Mann–Whitney U test, whereas paired observations were analyzed with the Wilcoxon signed-rank test. For data following an approximately normal distribution, values were expressed as mean ± standard deviation. Variance equality was examined through Levene’s test; if the assumption was met, intergroup differences were evaluated by the independent-samples t test, otherwise by Welch’s t test. Changes within the same group were analyzed with the paired-samples t test. Categorical variables were summarized as counts and proportions [n (%)]. Group comparisons were performed using the chi-square test, or Fisher’s exact test when the expected frequencies were small.

## Results

### Periodontal health indicators

During the observation period, four patients in the control group (three lost to follow-up, one withdrew voluntarily) and two patients in the combination group (both lost to follow-up) discontinued participation. Finally, data from 45 patients in the control group and 47 in the combination group were included in the final analysis. Following treatment, all parameters markedly improved in both groups, with the combination group exhibiting greater reductions in each indicator (*P* < 0.05) (**Table 2**).

**Table 2:** Comparison of periodontal health indicators between the two groups.

Time	Combination group (n = 47)	Control group (n = 45)	Z/t	*P*
Before treatment				
PD (mm)	5.80 (5.30, 6.05)	5.70 (5.30, 6.10)	0.973	0.331
AL (mm)	4.14 ± 0.57	4.14 ± 0.60	0.057	0.955
PLI (score)	2.33 ± 0.31	2.30 ± 0.33	0.46	0.647
SBI (score)	2.52 ± 0.28	2.48 ± 0.29	0.55	0.584
GI (score)	2.26 ± 0.28	2.23 ± 0.29	0.514	0.608
GCFV (μL)	0.28 ± 0.05	0.27 ± 0.05	0.719	0.474
After treatment				
PD (mm)	2.70 (2.40, 2.90)^a^	3.20 (2.80, 3.50)^a^	4.374	< 0.001
AL (mm)	2.84 ± 0.40^a^	3.45 ± 0.47^a^	6.699	< 0.001
PLI (score)	0.90 ± 0.20^a^	1.24 ± 0.25^a^	7.092	< 0.001
SBI (score)	0.72 ± 0.19^a^	1.18 ± 0.22^a^	10.915	< 0.001
GI (score)	0.68 ± 0.15^a^	1.06 ± 0.21^a^	9.914	< 0.001
GCFV (μL)	0.11 ± 0.02^a^	0.16 ± 0.03^a^	8.448	< 0.001

Compared with before treatment in the same group, ^a^*P* < 0.05.

### GCF inflammatory factors

Baseline concentrations of hs-CRP, TNF-α, IL-1β, and IL-6 were comparable between the two groups (*P* > 0.05). After therapy, all inflammatory markers decreased substantially in both groups, with lower post-treatment levels observed in the combination group relative to the control group (*P* < 0.05) (**Table 3**).

**Table 3:** Comparison of gingival crevicular fluid inflammatory factor levels between the two groups.

Time	Combination group (n=47)	Control group (n=45)	t	*P*
Before treatment				
hs-CRP (μmol/L)	22.46 ± 3.89	22.92 ± 3.85	0.566	0.572
TNF-α (pg/mL)	7.75 ± 1.52	7.42 ± 1.24	1.131	0.261
IL-1β (pg/mL)	14.85 ± 3.40	13.93 ± 2.92	1.39	0.168
IL-6 (pg/mL)	3.68 ± 0.54	3.86 ± 0.81	1.295	0.199
After treatment				
hs-CRP (μmol/L)	9.07 ± 2.20^a^	11.46 ± 2.71^a^	4.642	< 0.001
TNF-α (pg/mL)	3.51 ± 0.89^a^	4.88 ± 1.77^a^	4.628	< 0.001
IL-1β (pg/mL)	5.19 ± 1.34^a^	8.53 ± 2.39^a^	8.224	< 0.001
IL-6 (pg/mL)	1.07 ± 0.27^a^	1.83 ± 0.62^a^	7.529	< 0.001

Compared with before treatment in the same group, ^a^*P* < 0.05.

### Oxidative stress indicators

Prior to treatment, MDA, SOD, and GSH-Px levels showed no differences between groups (*P* > 0.05). After the 4-week treatment, MDA concentrations declined in both groups and were lower in the combination group, whereas SOD and GSH-Px activities increased more prominently in the combination group (*P* < 0.05) (**Table 4**).

**Table 4:** Comparison of oxidative stress indicator levels between the two groups.

Time	Combination group (n = 47)	Control group (n = 45)	t	*P*
Before treatment				
MDA (nmol/mL)	6.52 ± 1.04	6.48 ± 1.07	0.18	0.858
SOD (U/mL)	135.34 ± 11.55	136.01 ± 11.69	0.277	0.783
GSH-Px (U/mL)	68.52 ± 6.88	69.28 ± 7.12	0.522	0.603
After treatment				
MDA (nmol/mL)	3.17 ± 0.61^a^	4.72 ± 0.79^a^	10.588	< 0.001
SOD (U/mL)	165.47 ± 9.73^a^	152.69 ± 10.68^a^	6.004	< 0.001
GSH-Px (U/mL)	85.59 ± 5.73^a^	77.82 ± 6.26^a^	6.213	< 0.001

Compared with before treatment in the same group, ^a^*P* < 0.05.

### Chewing function and pain scores

At baseline, CFQ and VAS scores were similar between groups (*P* > 0.05). Following treatment, CFQ scores increased in both groups, with higher values in the combination group, while VAS scores decreased, with lower scores in the combination group (*P* < 0.05) (**Table 5**).

**Table 5:** Comparison of chewing function and pain levels between the two groups (score).

Time	Combination group (n = 47)	Control group (n = 45)	t/Z	*P*
Before treatment				
CFQ	17.85 ± 1.97	17.69 ± 1.89	0.403	0.688
VAS	5.00 (5.00, 6.00)	6.00 (5.00, 6.00)	0.669	0.504
After treatment				
CFQ	25.66 ± 1.72^a^	22.84 ± 1.83^a^	7.592	< 0.001
VAS	2.00 (1.00, 2.00)^a^	3.00 (2.00, 3.00)^a^	5.302	< 0.001

Compared with before treatment in the same group, ^a^*P* < 0.05.

### Clinical efficacy

Upon completion of treatment, the overall response rate was 97.87% (46/47) in the combination group, significantly exceeding 84.44% (38/45) in the control group (*P* < 0.05) (**Table 6**).

**Table 6:** Comparison of clinical efficacy between the two groups [n (%)].

Efficacy	Combination group (n = 47)	Control group (n = 45)	χ^2^	*P*
Remarkable	30 (63.83)	20 (44.44)	–	–
Effective	16 (34.04)	18 (40.00)	–	–
Ineffective	1 (2.13)	7 (15.56)	–	–
Total effective	46 (97.87)	38 (84.44)	*	0.029

*Fisher’s exact test.

### Adverse reactions

Throughout the study, adverse reactions occurred in 8.51% (4/47) of patients in the combination group and 4.26% (2/45) in the control group (*P* > 0.05) (**Table 7**).

**Table 7:** Comparison of adverse reactions between the two groups [n (%)].

Adverse reaction	Combination group (n = 47)	Control group (n = 45)	χ^2^	*P*
Oral mucosal irritation	2 (4.26)	1 (2.22)	–	–
Taste disturbance	1 (2.13)	0 (0.00)	–	–
Tooth sensitivity	1 (2.13)	1 (2.22)	–	–
Allergic responses	0 (0.00)	0 (0.00)	–	–
Total	4 (8.51)	2 (4.26)	*	0.677

*Fisher’s exact test.

## Discussion

This study provides novel evidence that combining Xipayi Gingival Solution (Xipayi Guyin Ye) with minocycline hydrochloride enhances clinical outcomes in patients with chronic periodontitis. Compared with minocycline monotherapy, the combined therapy improves periodontal clinical indicators, inflammation levels, oxidative stress status, as well as chewing function and pain perception in patients with chronic periodontitis, without increasing adverse reactions. These findings demonstrate that integrating a traditional Uyghur medicine preparation with a modern antibiotic produces synergistic antibacterial, anti-inflammatory, and antioxidant effects, representing an innovative adjunctive strategy for periodontal treatment.

The present study showed that following treatment, the combination group outperformed the control group in core periodontal indicators such as PD, AL, PLI, and SBI, indicating that the combination of Xipayi Gingival Solution and minocycline hydrochloride in the treatment of chronic periodontitis can improve patients’ periodontal health status. Minocycline hydrochloride itself has been proven effective in reducing key periodontal pathogens and improving clinical indicators (Arnett et al., 2023). The core polyphenolic component of Xipayi Gingival Solution, tannin (tannic acid), has been confirmed to possess anti-inflammatory, antibacterial, and antioxidant properties that can serve as an effective adjunct to basic periodontal treatment (Malcangi et al., 2025). The combination of the two may produce a synergistic effect through the targeted antibacterial action of minocycline and the broad-spectrum biofilm inhibition and local inflammation reduction by the polyphenolic substances in Xipayi Gingival Solution. The findings of this study are consistent with the principle of local antibiotic potentiation. A 2024 review pointed out that for periodontitis patients with diabetes, adding local adjunctive drugs (such as statins, metformin, antibiotics) to non-surgical treatment can more significantly reduce periodontal pocket depth and increase clinical attachment level in the short and medium term compared to basic treatment alone (Lin et al., 2024).

Further exploration revealed that the combination group outperformed the control group in reducing inflammatory factors such as hs-CRP, TNF-α, IL-1β, and IL-6, as well as improving oxidative stress indicators (decreased MDA, increased SOD, and GSH-Px). Xipayi Gingival Solution may inhibit inflammatory signaling pathways through its polyphenolic components (Malcangi et al., 2025), reducing the release of pro-inflammatory factors while scavenging free radicals and enhancing the activity of endogenous antioxidant enzymes (Chen et al., 2020; Yang et al., 2022; Lopez-Valverde et al., 2023), thereby complementing the anti-inflammatory effects of minocycline (Arnett et al., 2023; Zhang et al., 2024) and more comprehensively improving the periodontal microenvironment, breaking the vicious cycle of inflammation-oxidative stress (Dai et al., 2022; Chang et al., 2024). Chen et al. demonstrated that the combination of Xipayi mouth rinse and minocycline in the treatment of localized aggressive periodontitis significantly improved periodontal gingival status and reduced inflammatory factor levels (Chen et al., 2020). Notably, the core identified components of Xipayi Gingival Solution include polyphenolic compounds such as gallic acid (Chen et al., 2024). Polyphenols are widely recognized as potent free radical scavengers (Arunachalam et al., 2022; Tossetta et al., 2024). One study on similar plant polyphenols suggests that these components may upregulate the activity of endogenous antioxidant enzymes such as SOD and GSH-Px by activating the NRF2 pathway, which aligns with the observed changes in antioxidant enzyme levels in this study (Tossetta et al., 2024).

This study also found that the combination group outperformed the control group in improving chewing function scores and reducing pain scores, indicating that the combined therapy can more effectively improve patients’ chewing function and alleviate pain symptoms. Clinical evidence supports that Xipayi-based adjunctive therapy can promote gingival healing and enhance chewing efficiency (Mai et al., 2021), while minocycline-containing regimens effectively alleviate periodontal pain and discomfort (Chang et al., 2024). These findings collectively support the observed improvements in functional recovery and patient comfort in the combination group.

Regarding clinical outcomes, the overall effective rate in the combination group was 97.87%, significantly higher than 84.44% in the minocycline-only group, reflecting the advantage of combined therapy. The combination of Xipayi Gingival Solution and minocycline hydrochloride theoretically constitutes a complementary “antibacterial” and “anti-inflammatory/antioxidant” dual mechanism, while clinically achieving a synergistic effect of “efficacy enhancement” and “functional recovery.”

In conclusion, Xipayi Gingival Solution combined with minocycline improves periodontal health, reduces inflammatory and oxidative stress markers, restores masticatory function, and achieves superior clinical outcomes compared to minocycline alone, without increasing adverse reactions, offering a safe and effective strategy for chronic periodontitis management. However, as a single-center study with a limited sample size and short follow-up period, the generalizability of the results of this study is limited, and it fails to elucidate the mechanism of action of the combined therapy at the oral microbiome and molecular pathway levels. Future research should conduct multi-center, large-sample, long-term studies and utilize omics technologies to further validate its long-term efficacy and precisely dissect its molecular mechanisms for improving the periodontal microenvironment by regulating host immune-inflammatory pathways and antioxidant defense systems, thereby providing more solid evidence for the optimization and promotion of treatment regimens for chronic periodontitis.

## Data Availability

The raw data supporting the conclusions of this article will be made available by the authors, without undue reservation.
